# Supplementary figures and images for: PAAD: Panelization algorithm for architectural designs
Source: PLoS One. 2024 Jun 11;19(6):e0303646. doi: 10.1371/journal.pone.0303646 (PMC11166312; doi:10.1371/journal.pone.0303646)

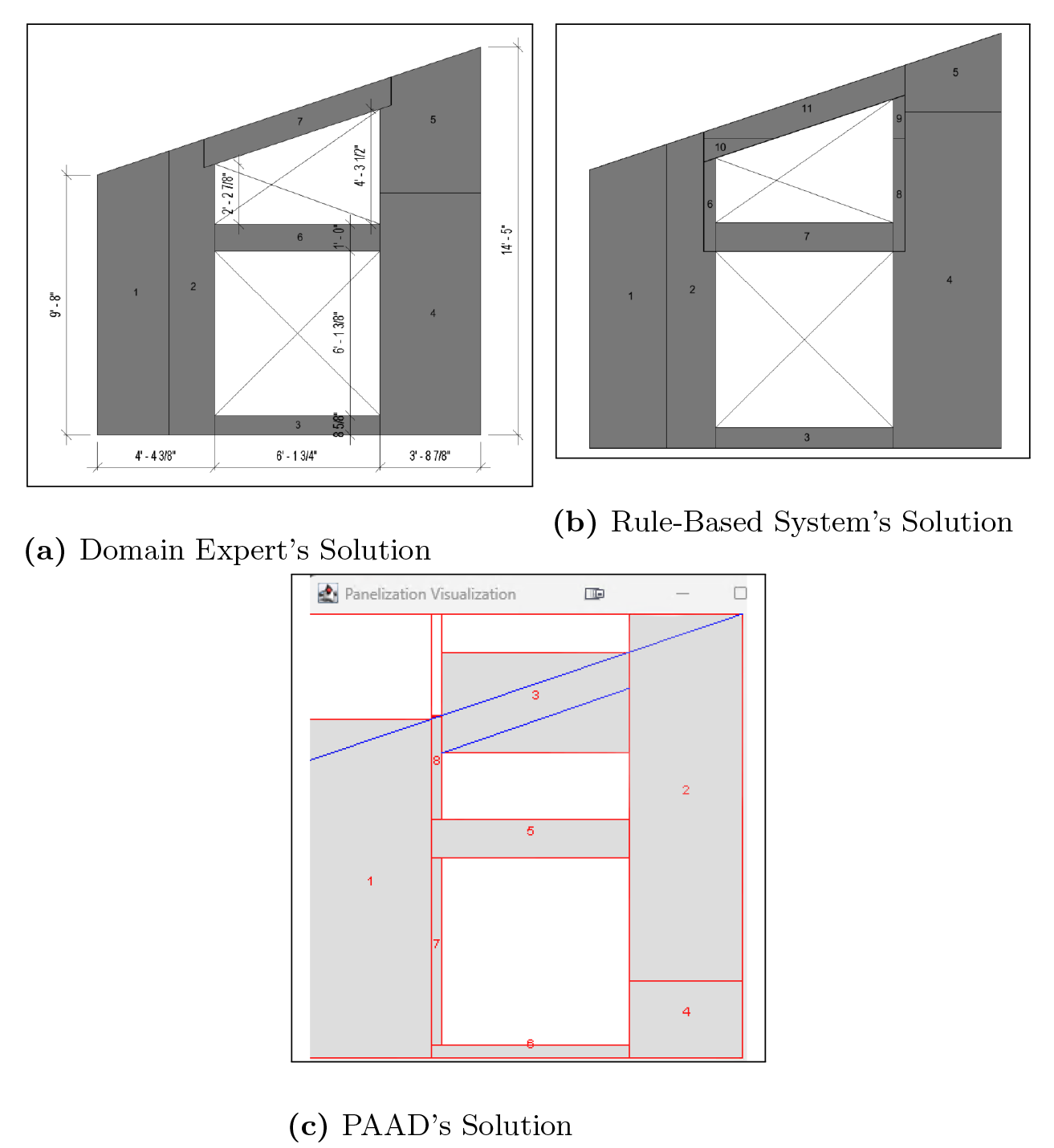

Supplement: S1 Fig — The results of automated scenario 1, where (a) presents the domain expert’s solution, (b) shows the rule-based system’s output, and (c) shows PAAD’s solution. (TIF) [file pone.0303646.s001.tif]

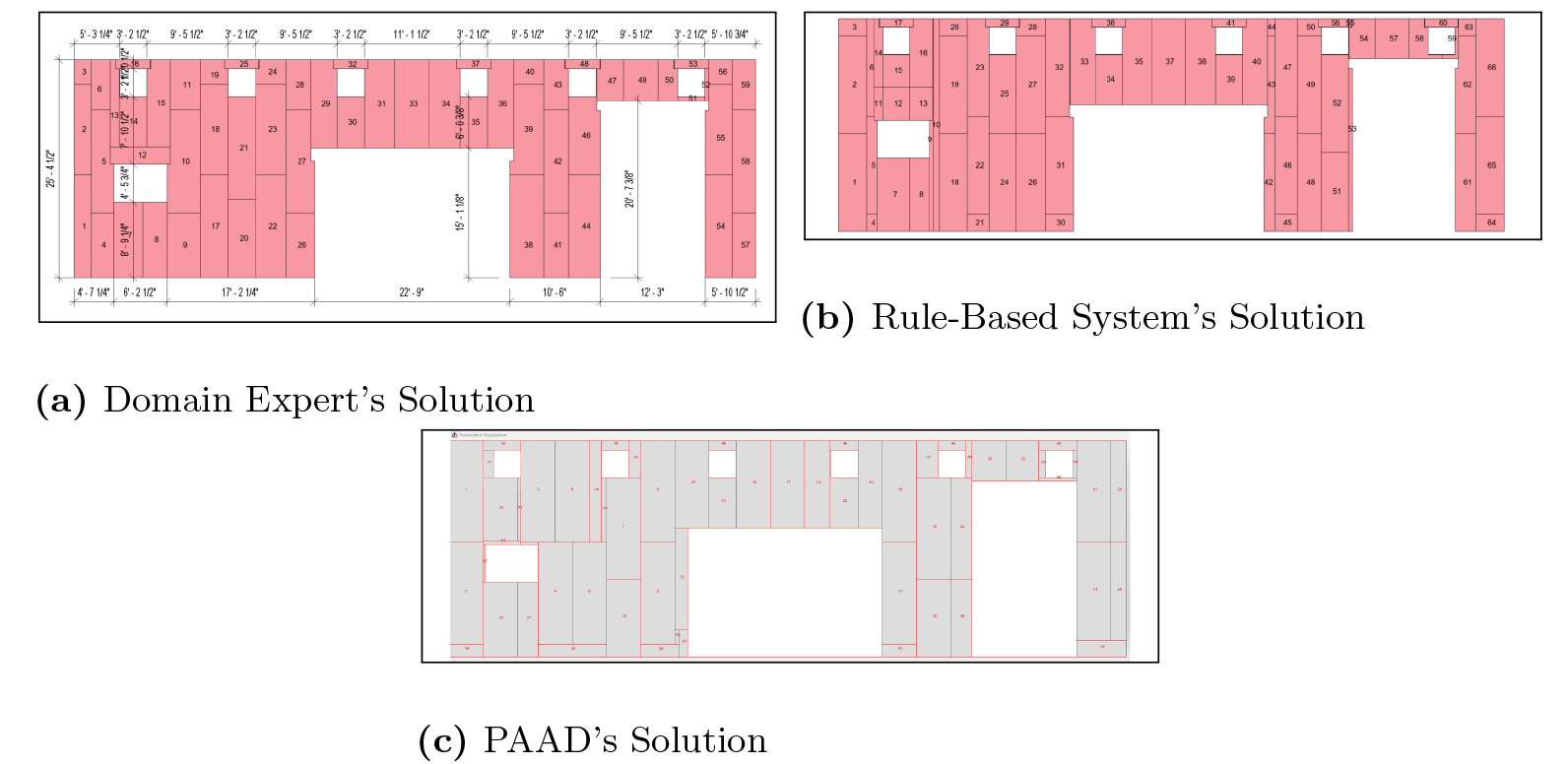

Supplement: S2 Fig — The results of automated scenario 2, where (a) presents the domain expert’s solution, (b) shows the rule-based system’s output, and (c) shows PAAD’s solution. (TIF) [file pone.0303646.s002.tif]

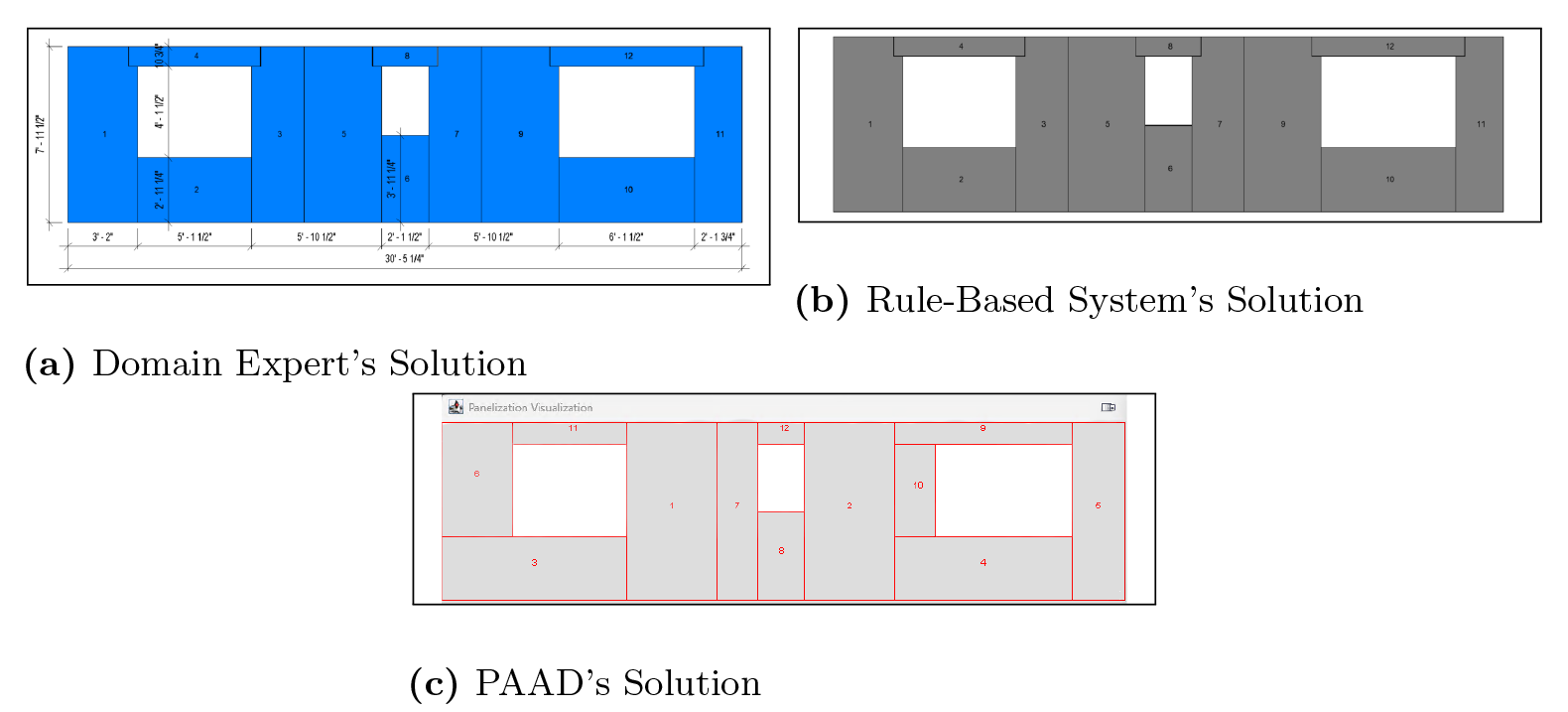

Supplement: S3 Fig — The results of automated scenario 3, where (a) presents the domain expert’s solution, (b) shows the rule-based system’s output, and (c) shows PAAD’s solution. (TIF) [file pone.0303646.s003.tif]

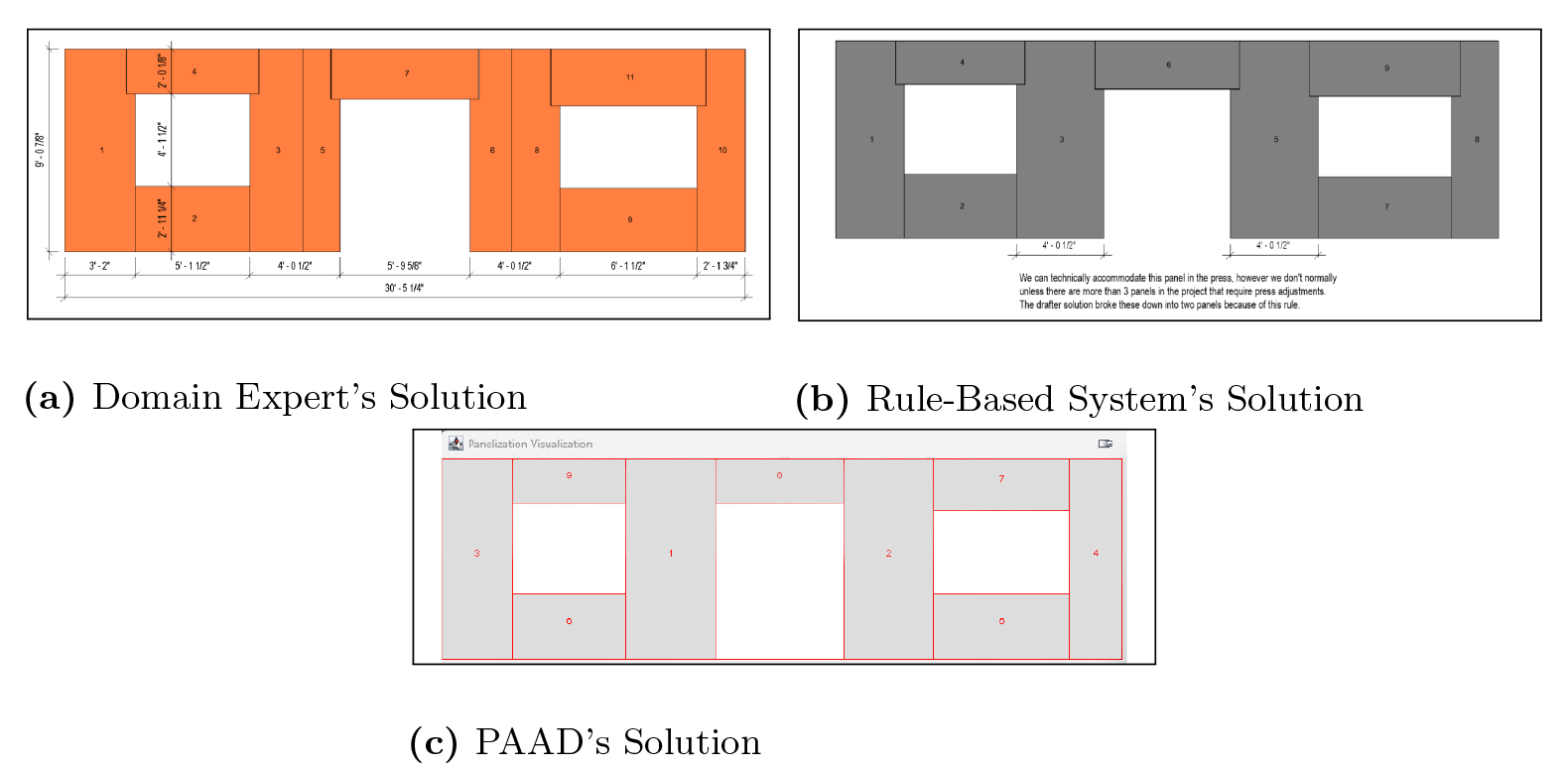

Supplement: S4 Fig — The results of automated scenario 4, where (a) presents the domain expert’s solution, (b) shows the rule-based system’s output, and (c) shows PAAD’s solution. (TIF) [file pone.0303646.s004.tif]

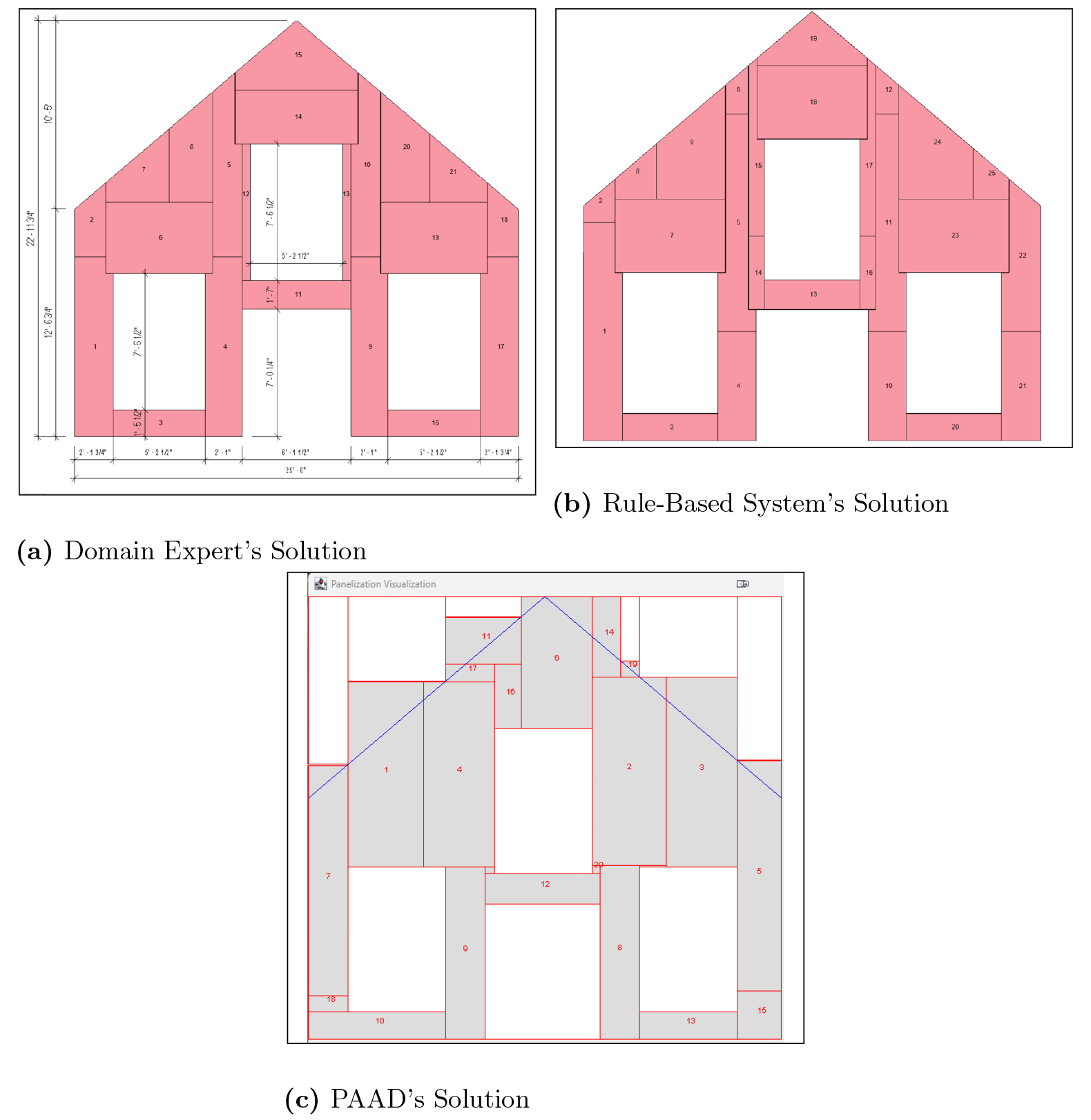

Supplement: S5 Fig — The results of automated scenario 5, where (a) presents the domain expert’s solution, (b) shows the rule-based system’s output, and (c) shows PAAD’s solution. (TIF) [file pone.0303646.s005.tif]

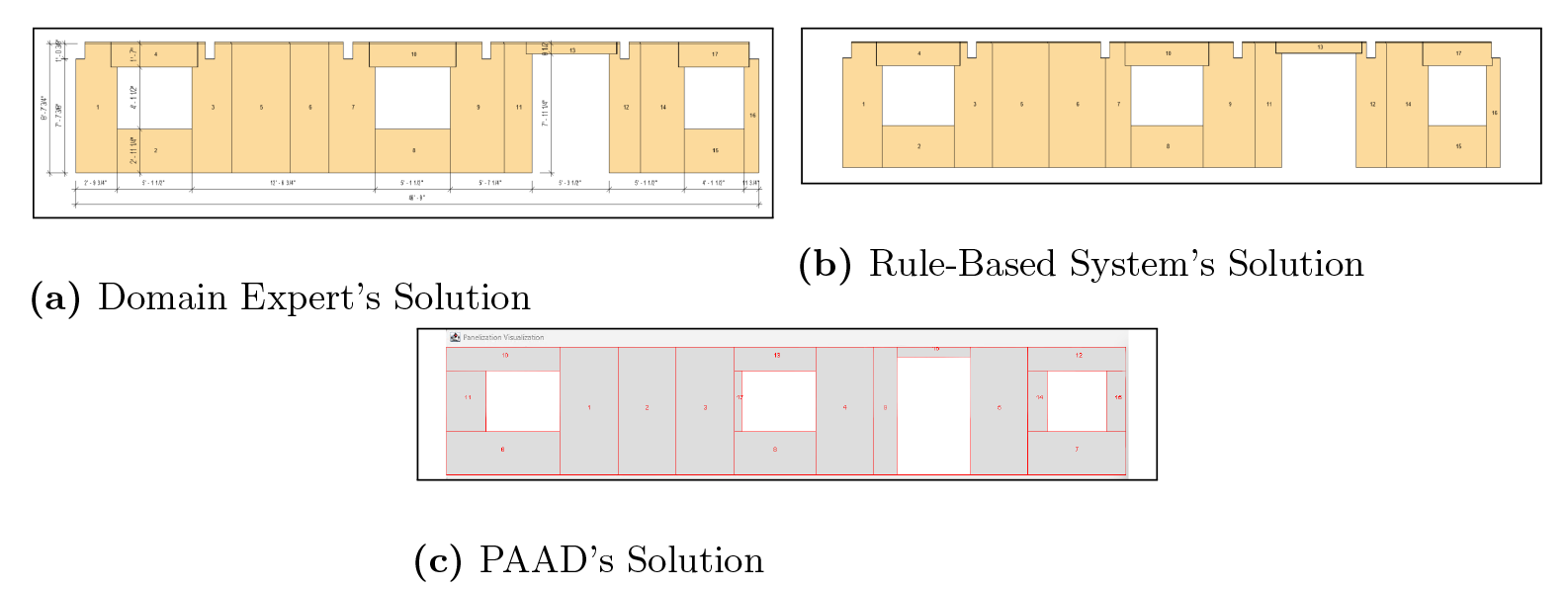

Supplement: S6 Fig — The results of automated scenario 6, where (a) presents the domain expert’s solution, (b) shows the rule-based system’s output, and (c) shows PAAD’s solution. (TIF) [file pone.0303646.s006.tif]

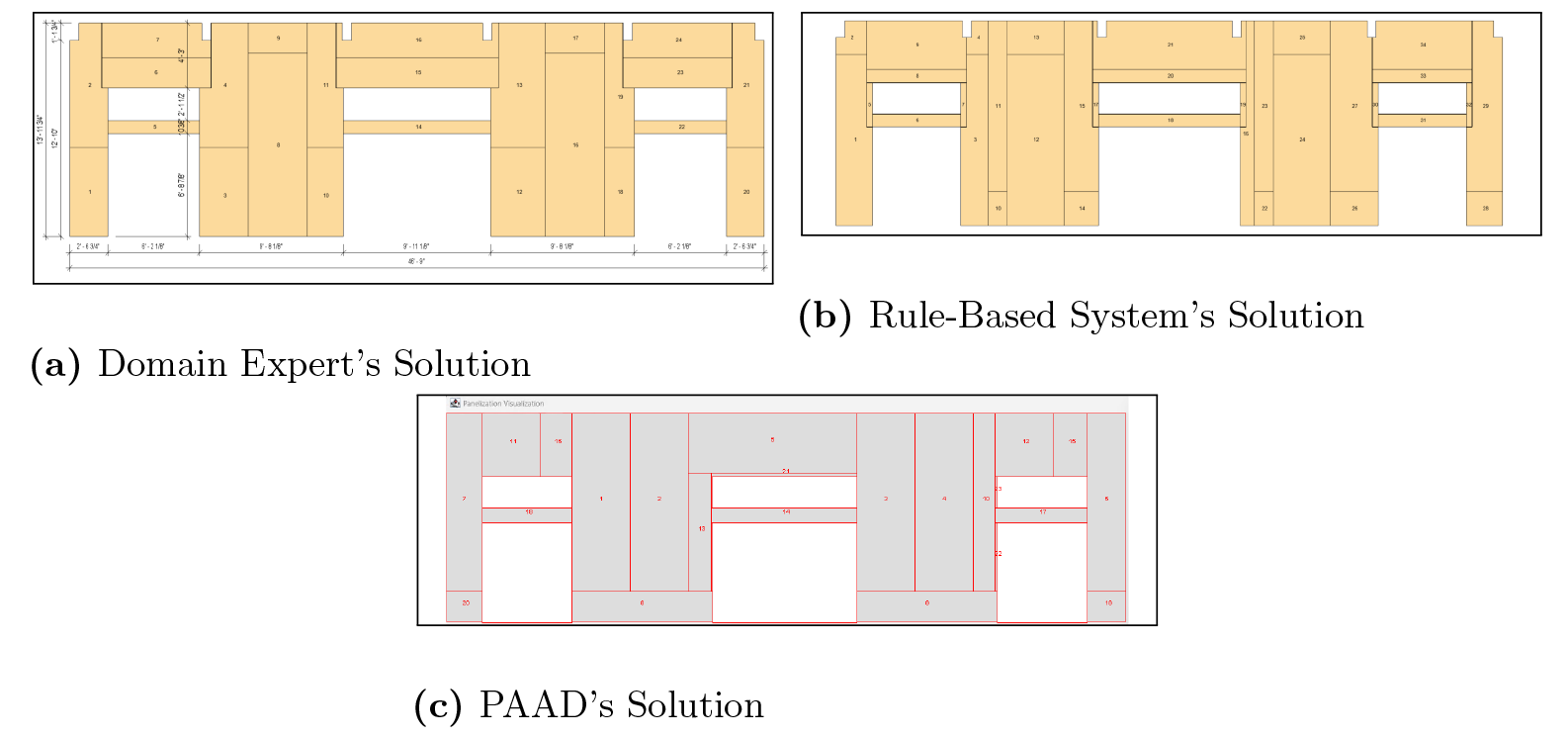

Supplement: S7 Fig — The results of automated scenario 7, where (a) presents the domain expert’s solution, (b) shows the rule-based system’s output, and (c) shows PAAD’s solution. (TIF) [file pone.0303646.s007.tif]

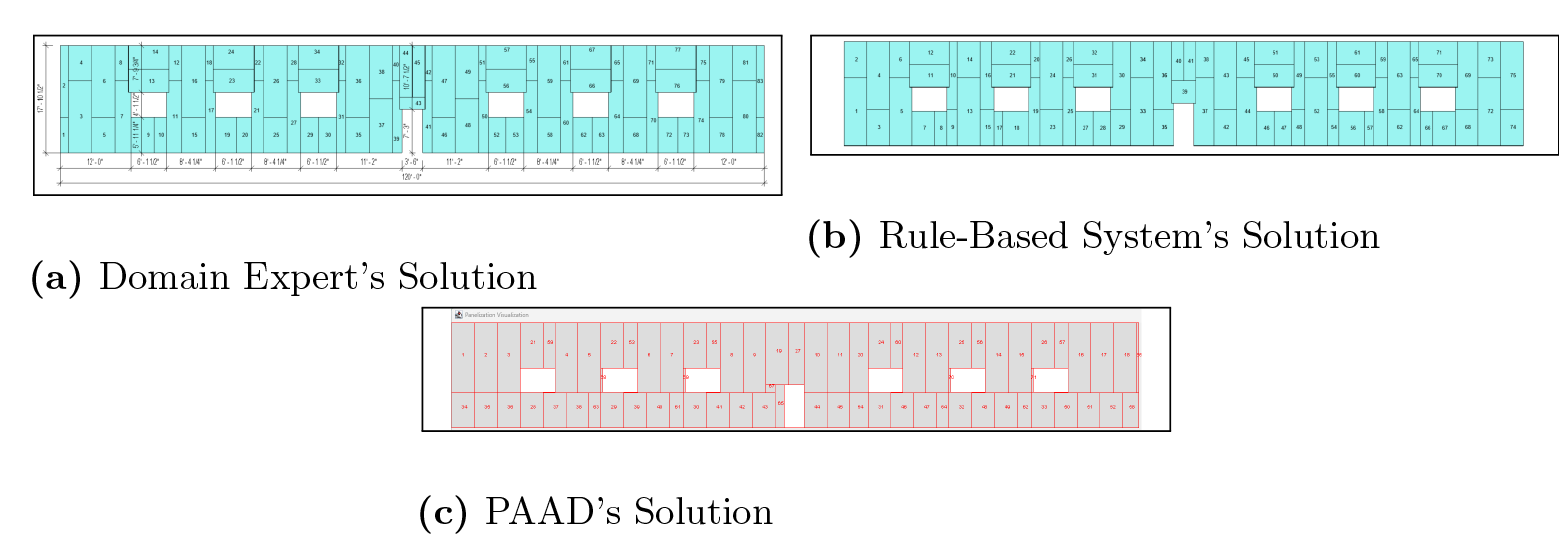

Supplement: S8 Fig — The results of automated scenario 8, where (a) presents the domain expert’s solution, (b) shows the rule-based system’s output, and (c) shows PAAD’s solution. (TIF) [file pone.0303646.s008.tif]

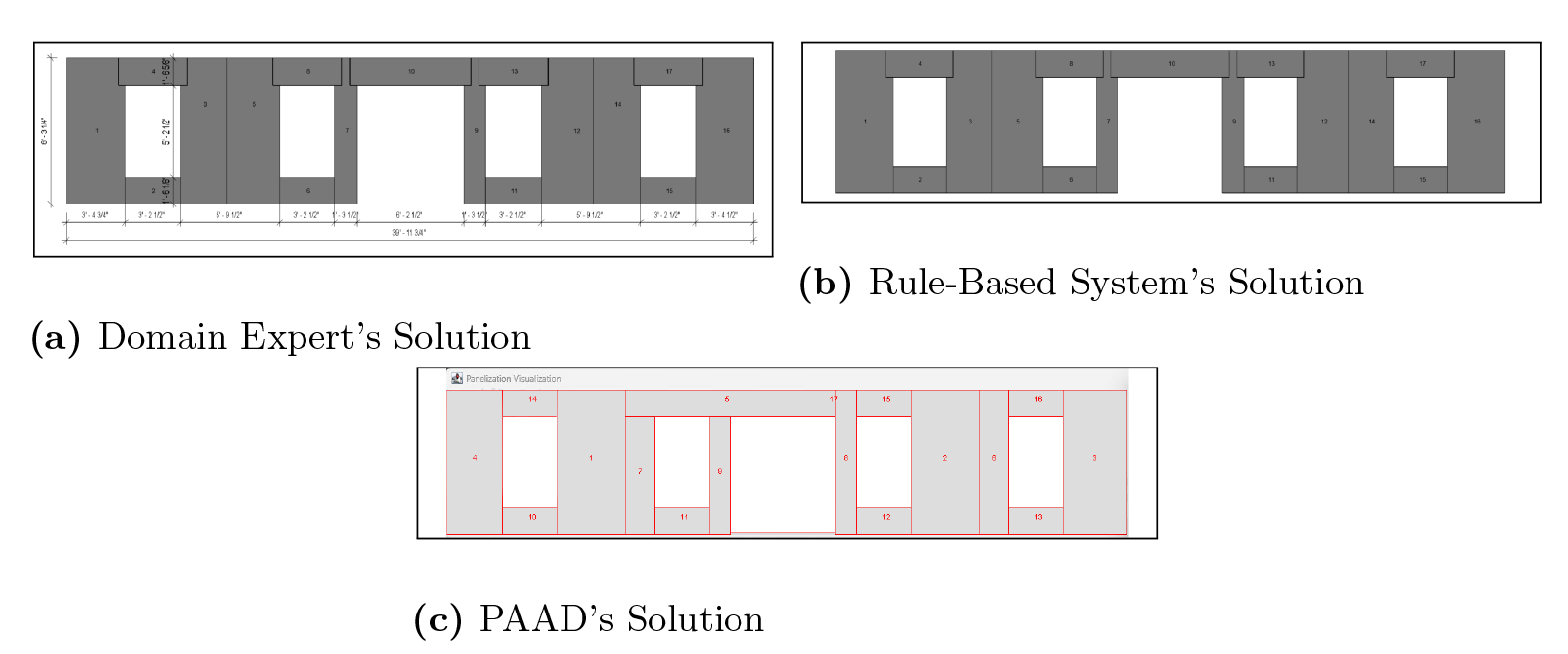

Supplement: S9 Fig — The results of automated scenario 9, where (a) presents the domain expert’s solution, (b) shows the rule-based system’s output, and (c) shows PAAD’s solution. (TIF) [file pone.0303646.s009.tif]

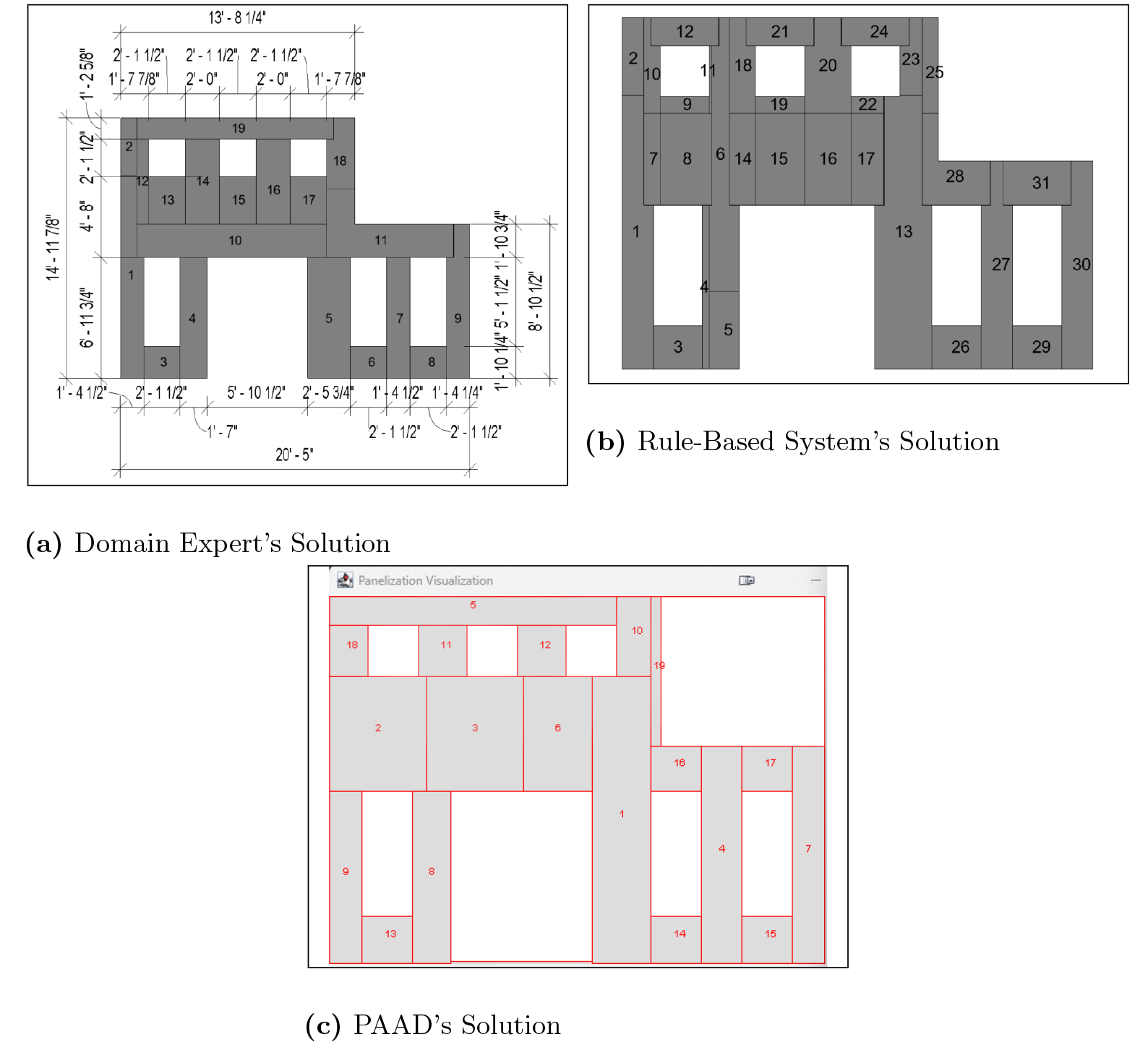

Supplement: S10 Fig — The results of automated scenario 11, where (a) presents the domain expert’s solution, (b) shows the rule-based system’s output, and (c) shows PAAD’s solution. (TIF) [file pone.0303646.s010.tif]

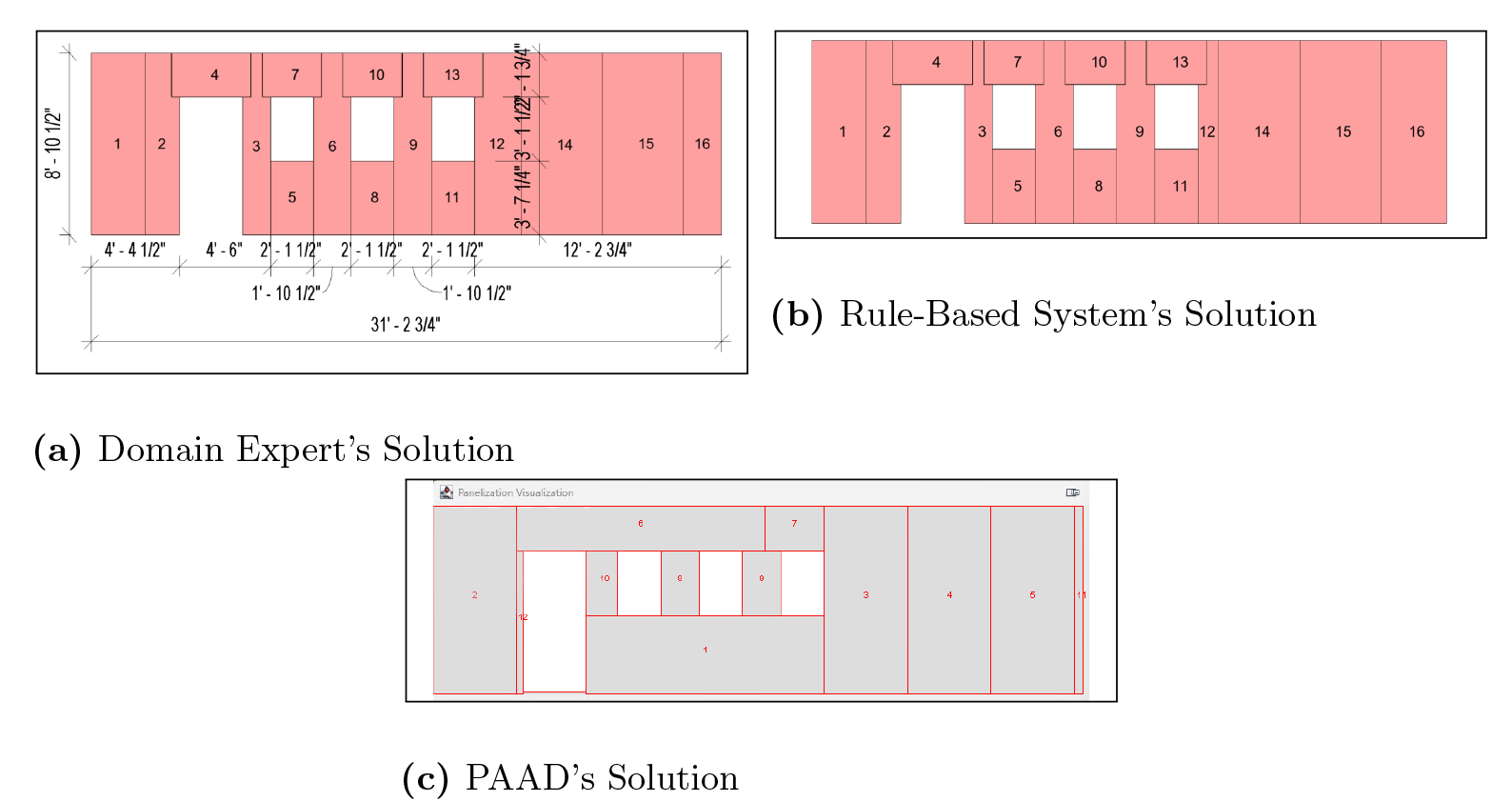

Supplement: S11 Fig — The results of automated scenario 12, where (a) presents the domain expert’s solution, (b) shows the rule-based system’s output, and (c) shows PAAD’s solution. (TIF) [file pone.0303646.s011.tif]

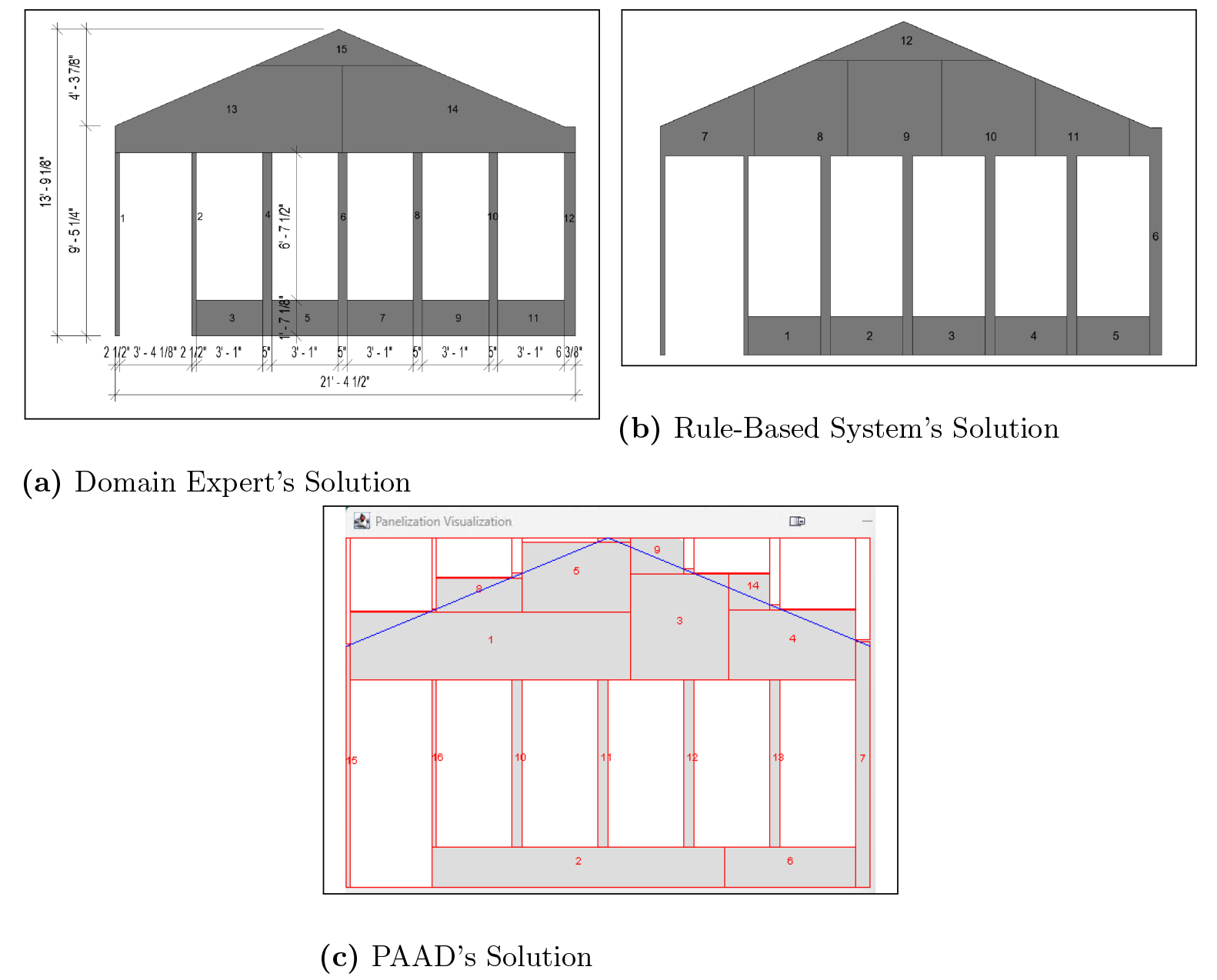

Supplement: S12 Fig — The results of automated scenario 13, where (a) presents the domain expert’s solution, (b) shows the rule-based system’s output, and (c) shows PAAD’s solution. (TIF) [file pone.0303646.s012.tif]

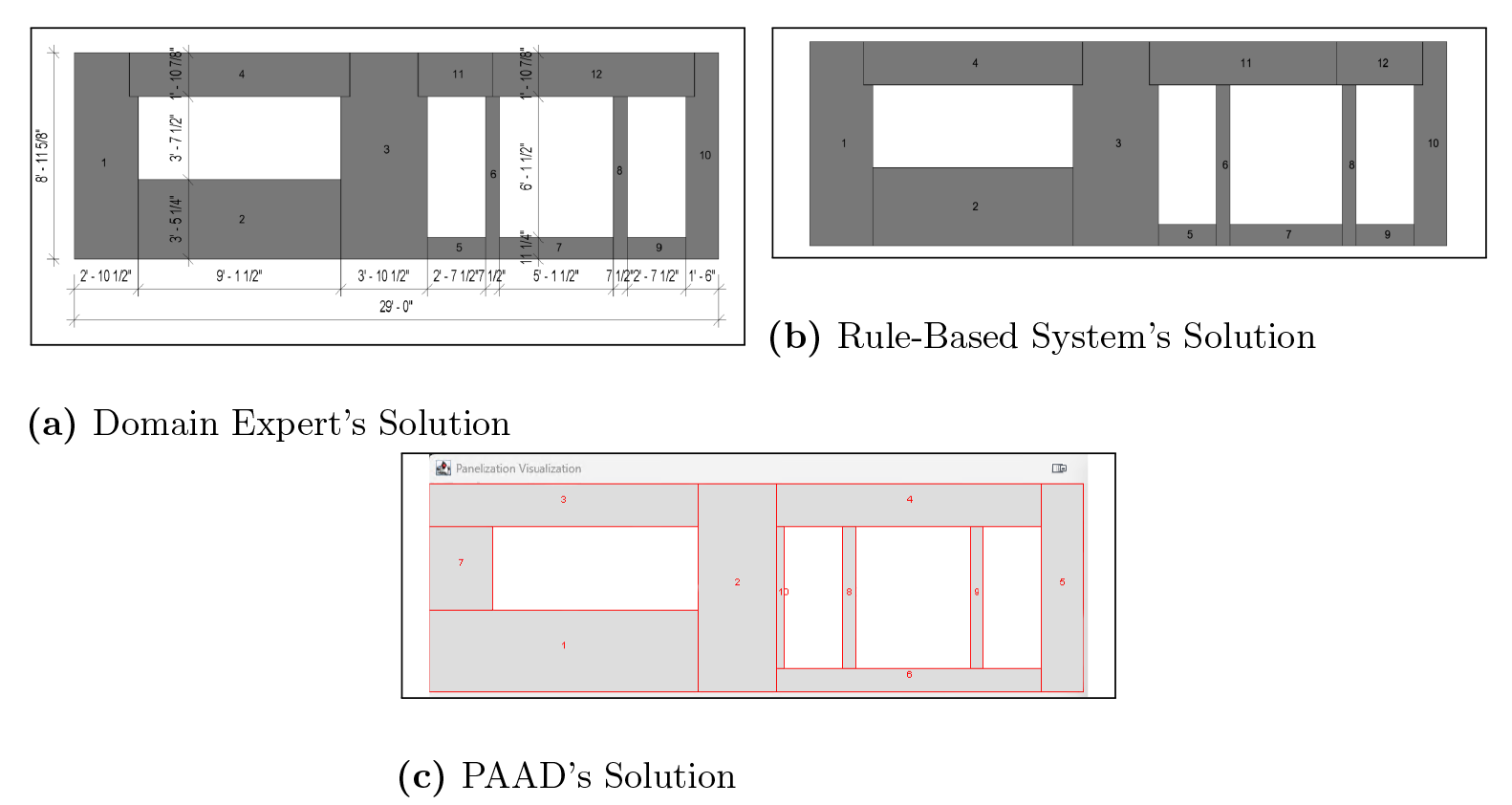

Supplement: S13 Fig — The results of automated scenario 14, where (a) presents the domain expert’s solution, (b) shows the rule-based system’s output, and (c) shows PAAD’s solution. (TIF) [file pone.0303646.s013.tif]

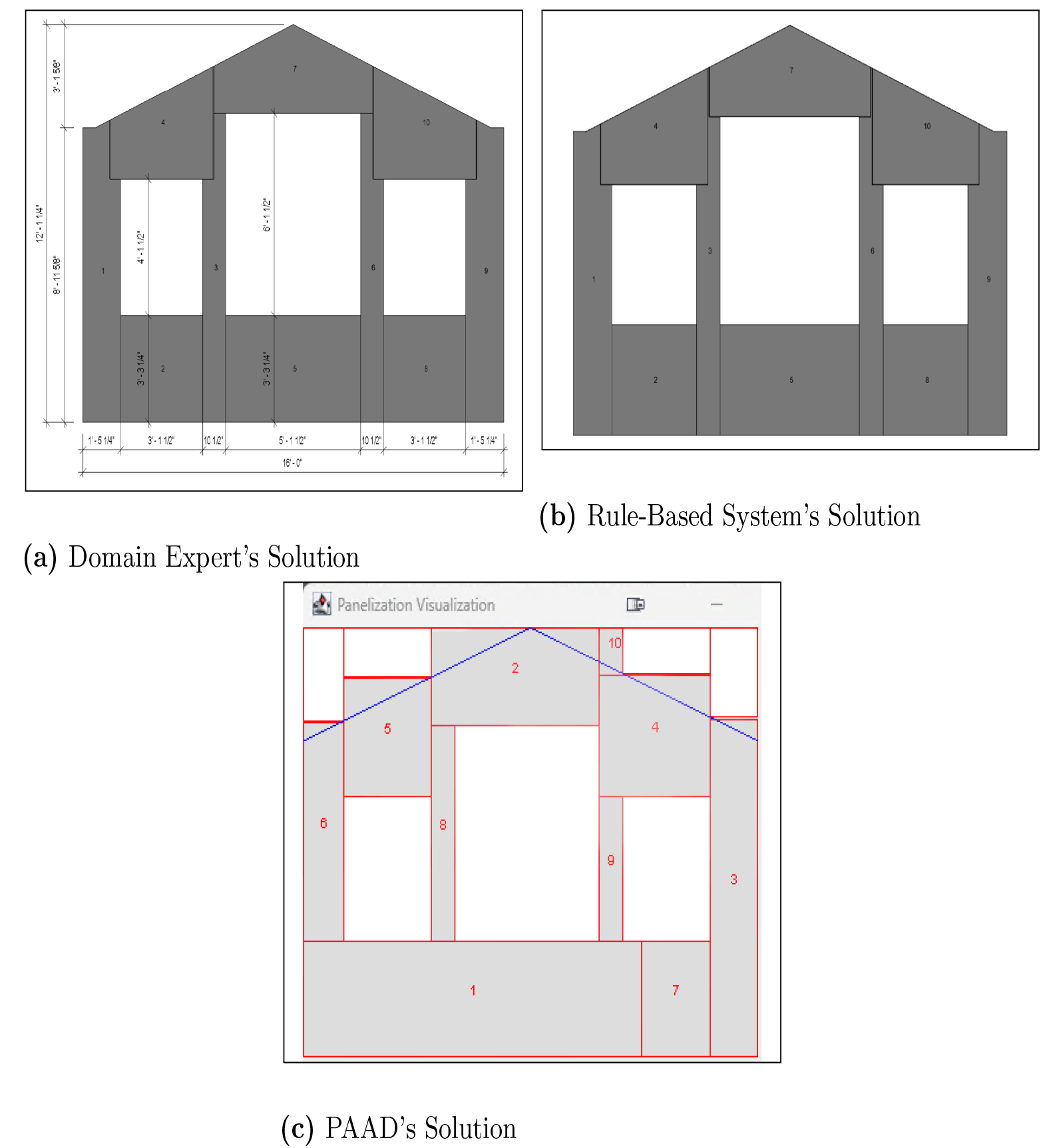

Supplement: S14 Fig — The results of automated scenario 15, where (a) presents the domain expert’s solution, (b) shows the rule-based system’s output, and (c) shows PAAD’s solution. (TIF) [file pone.0303646.s014.tif]

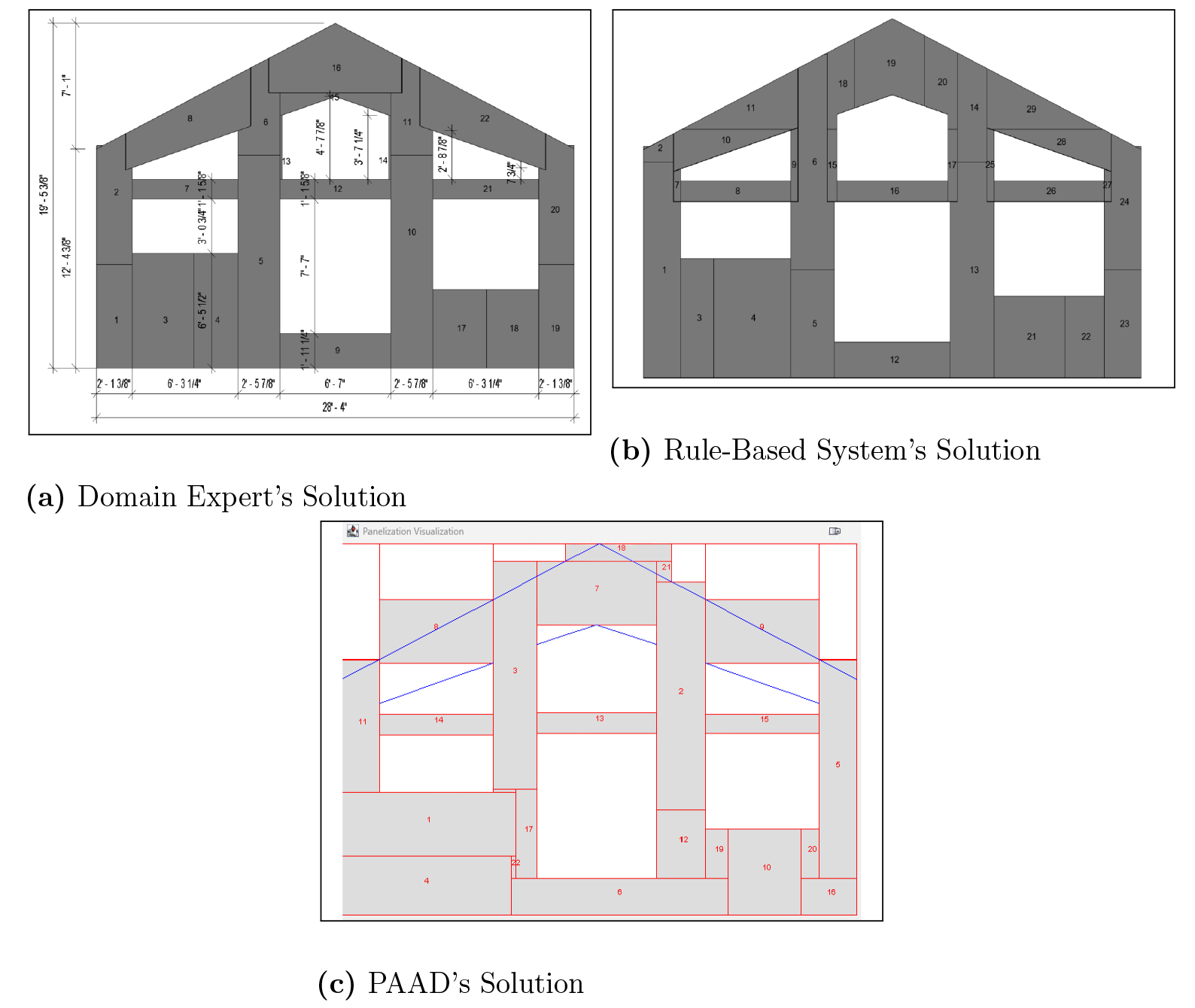

Supplement: S15 Fig — The results of automated scenario 16, where (a) presents the domain expert’s solution, (b) shows the rule-based system’s output, and (c) shows PAAD’s solution. (TIF) [file pone.0303646.s015.tif]

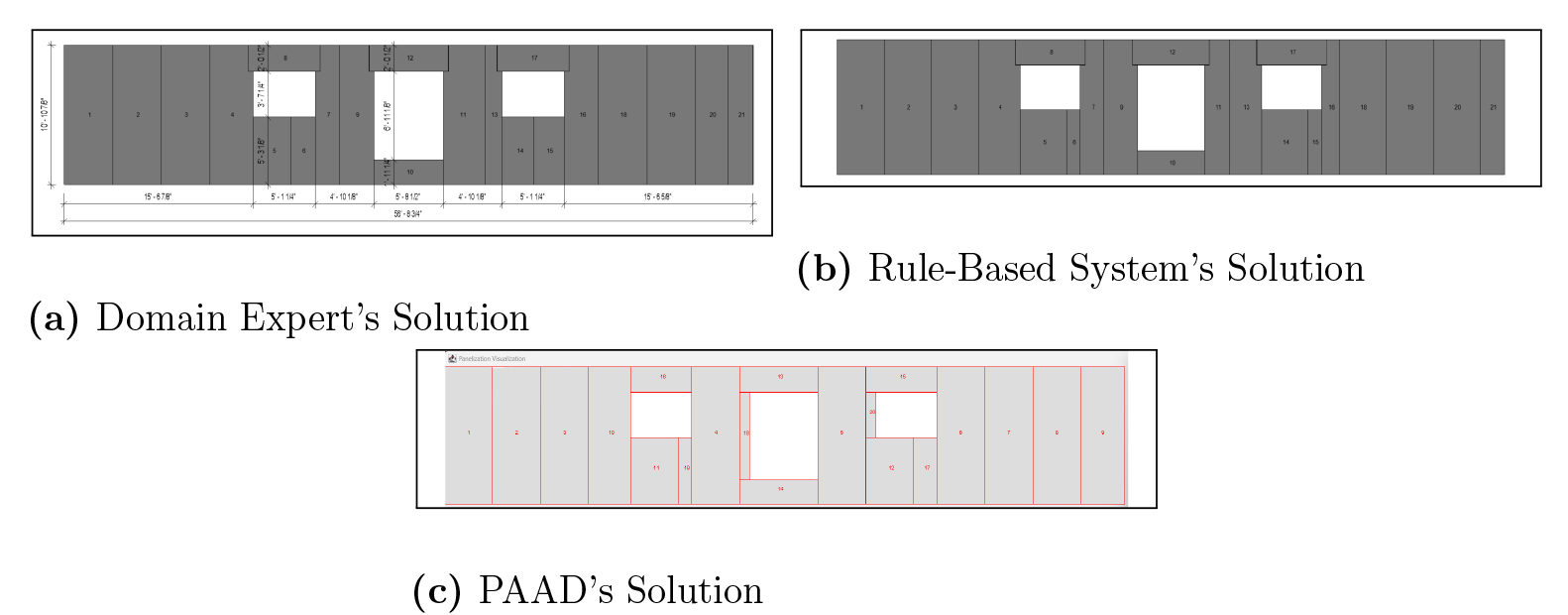

Supplement: S16 Fig — The results of automated scenario 17, where (a) presents the domain expert’s solution, (b) shows the rule-based system’s output, and (c) shows PAAD’s solution. (TIF) [file pone.0303646.s016.tif]

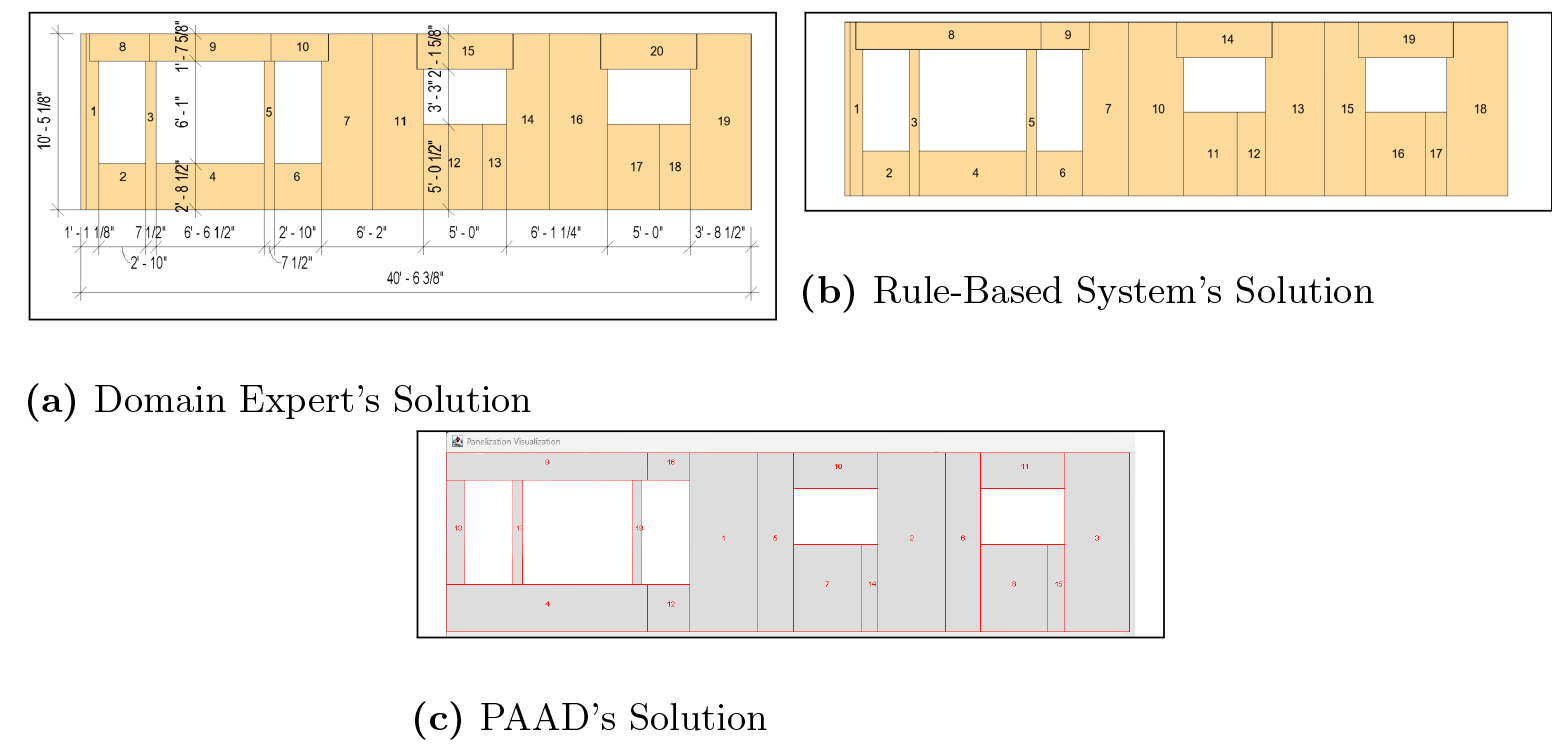

Supplement: S17 Fig — The results of automated scenario 18, where (a) presents the domain expert’s solution, (b) shows the rule-based system’s output, and (c) shows PAAD’s solution. (TIF) [file pone.0303646.s017.tif]

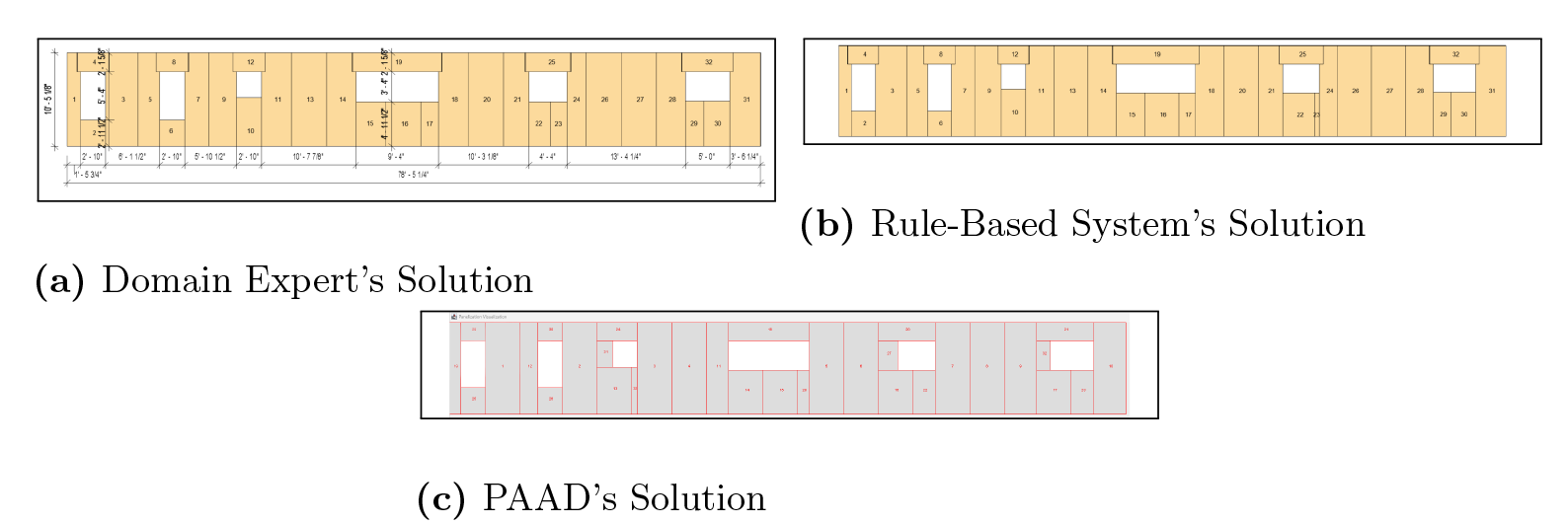

Supplement: S18 Fig — The results of automated scenario 19, where (a) presents the domain expert’s solution, (b) shows the rule-based system’s output, and (c) shows PAAD’s solution. (TIF) [file pone.0303646.s018.tif]

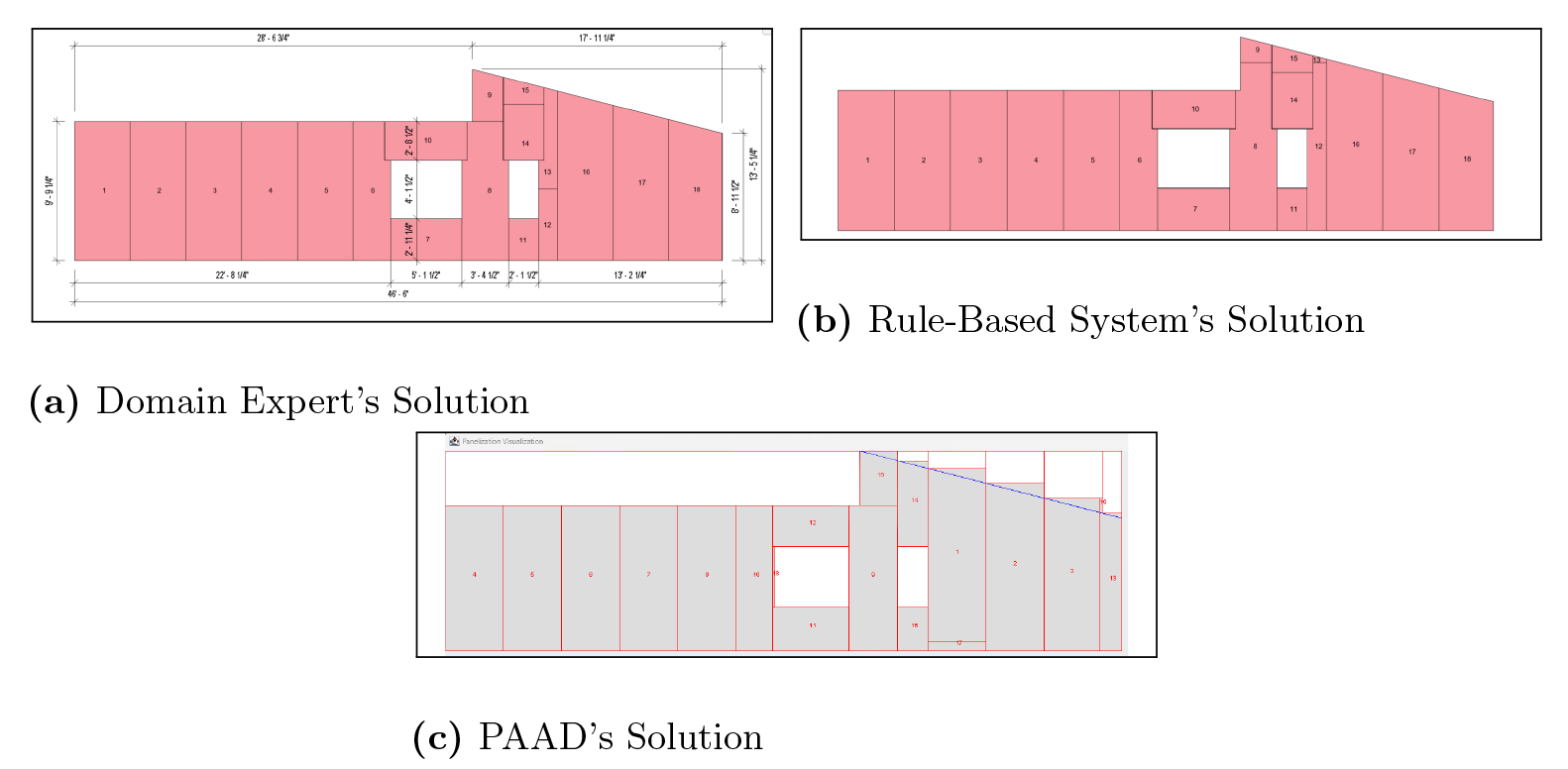

Supplement: S19 Fig — The results of automated scenario 21, where (a) presents the domain expert’s solution, (b) shows the rule-based system’s output, and (c) shows PAAD’s solution. (TIF) [file pone.0303646.s019.tif]

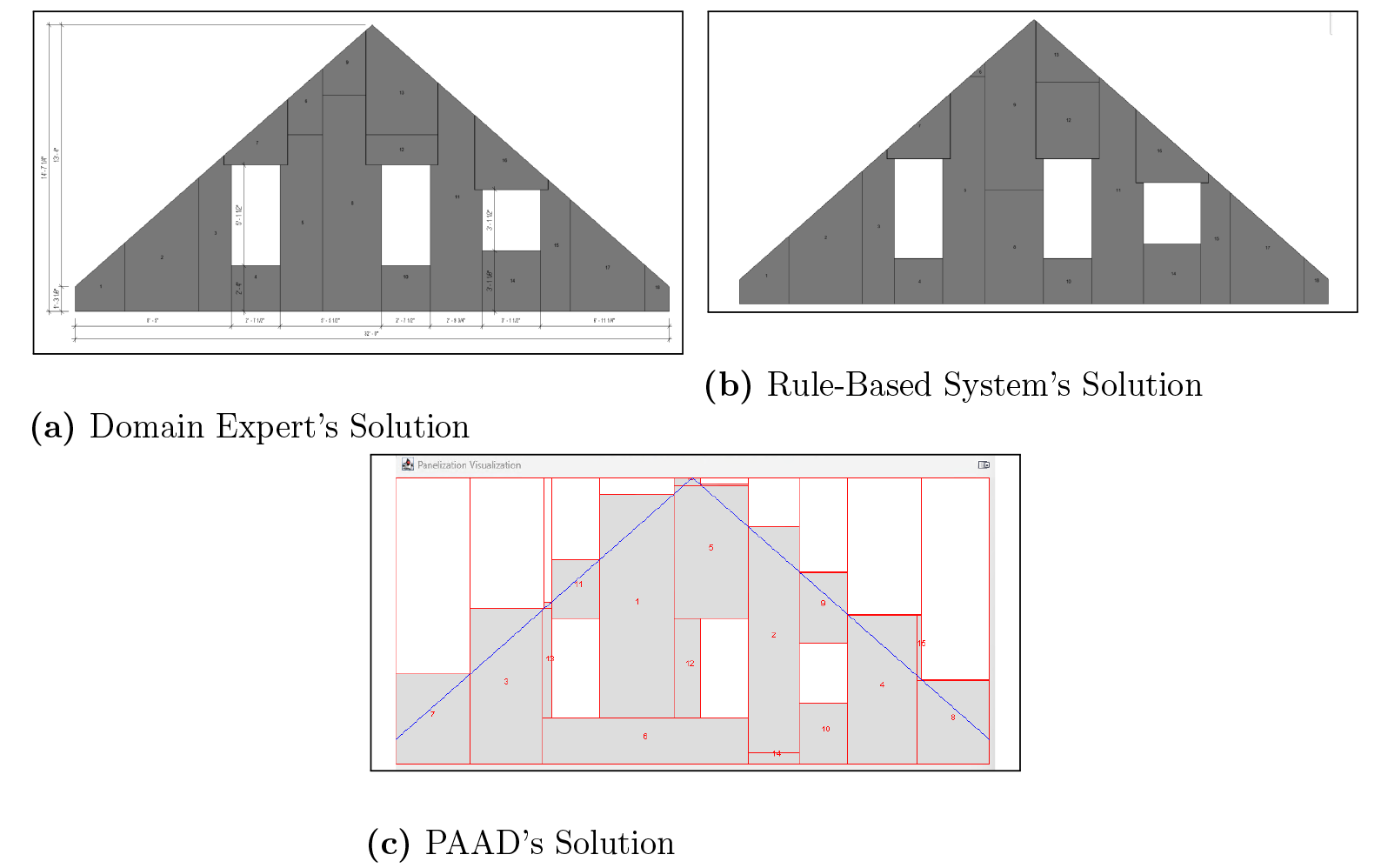

Supplement: S20 Fig — The results of automated scenario 22, where (a) presents the domain expert’s solution, (b) shows the rule-based system’s output, and (c) shows PAAD’s solution. (TIF) [file pone.0303646.s020.tif]

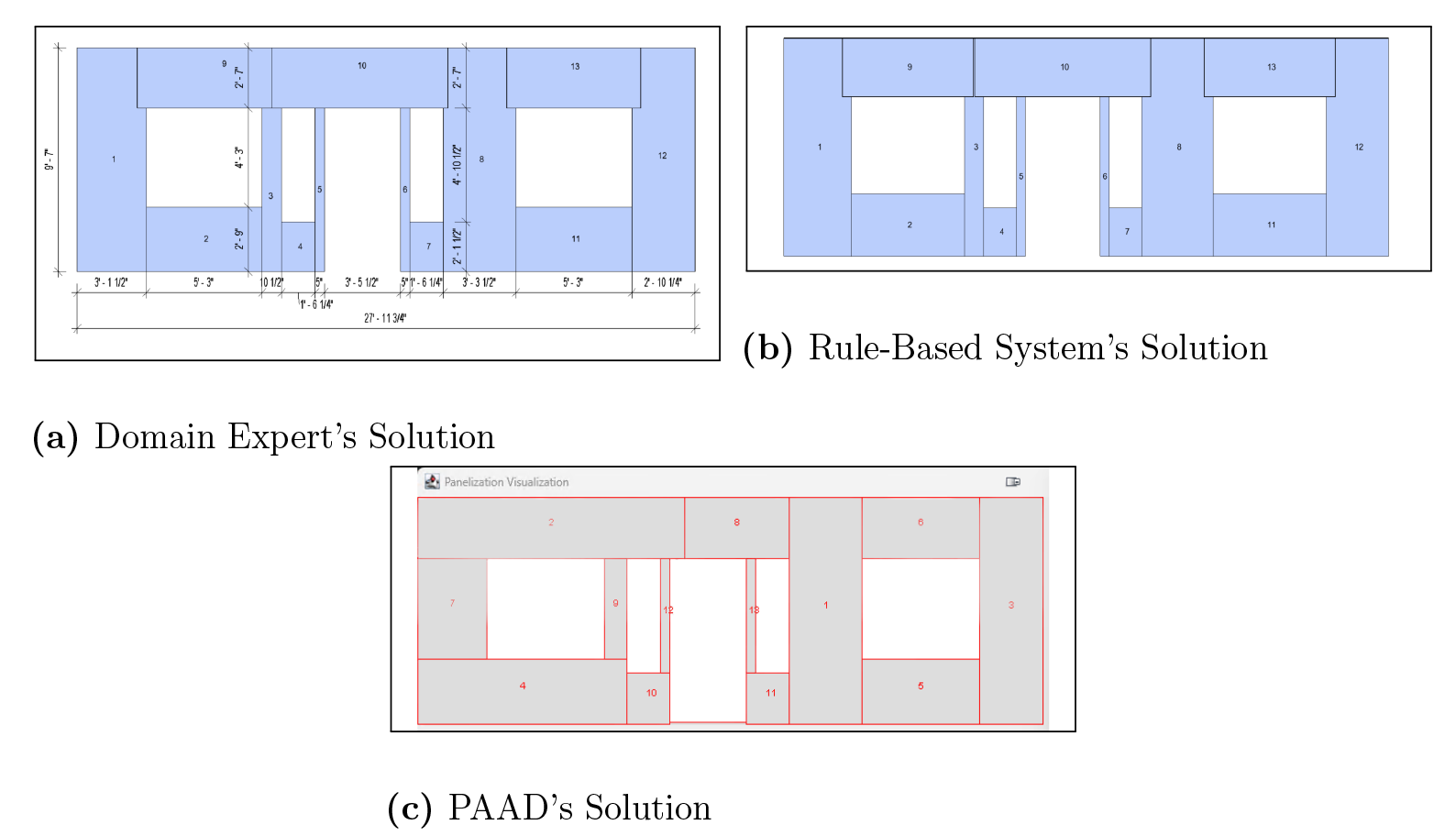

Supplement: S21 Fig — The results of automated scenario 23, where (a) presents the domain expert’s solution, (b) shows the rule-based system’s output, and (c) shows PAAD’s solution. (TIF) [file pone.0303646.s021.tif]

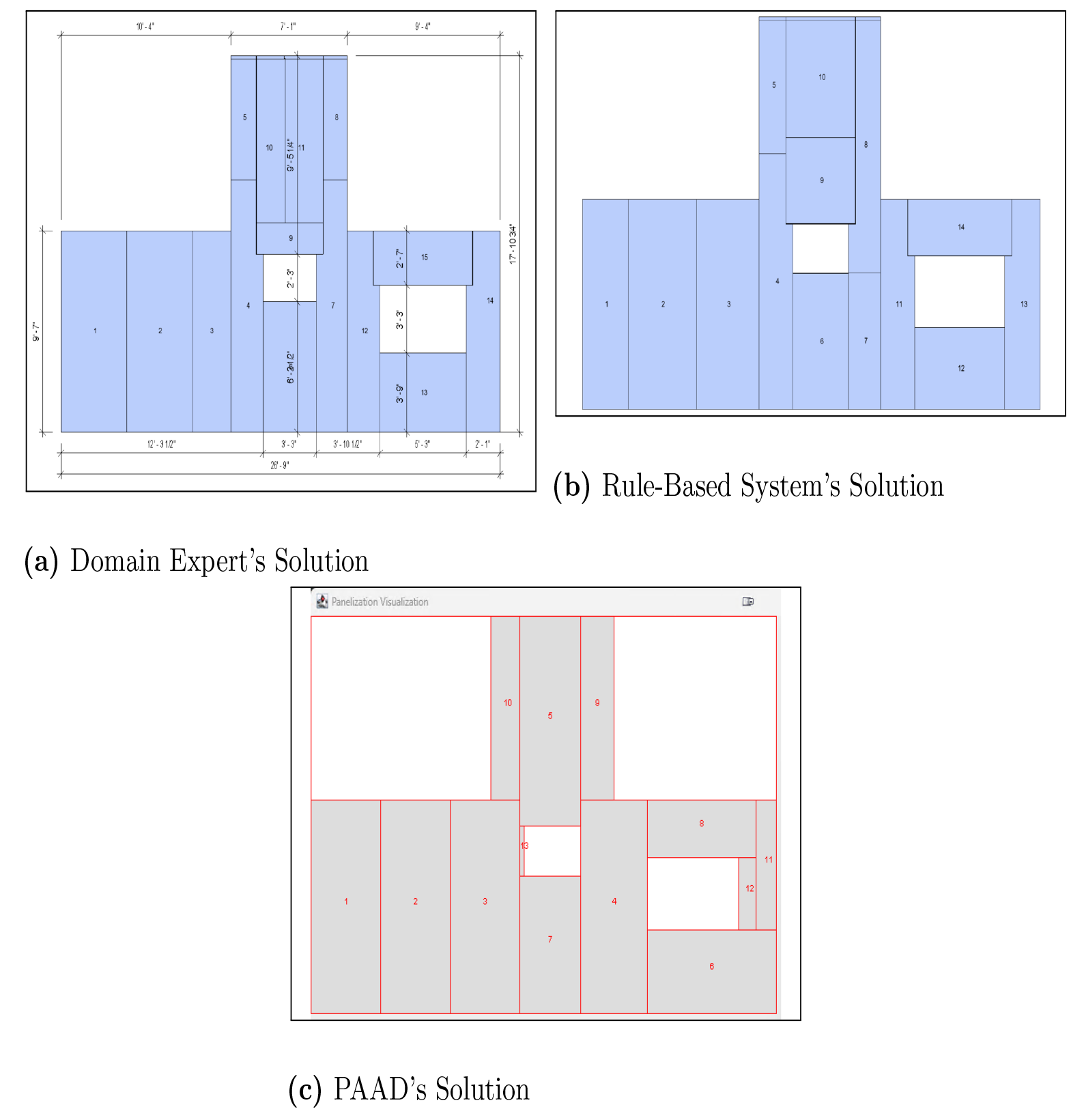

Supplement: S22 Fig — The results of automated scenario 24, where (a) presents the domain expert’s solution, (b) shows the rule-based system’s output, and (c) shows PAAD’s solution. (TIF) [file pone.0303646.s022.tif]

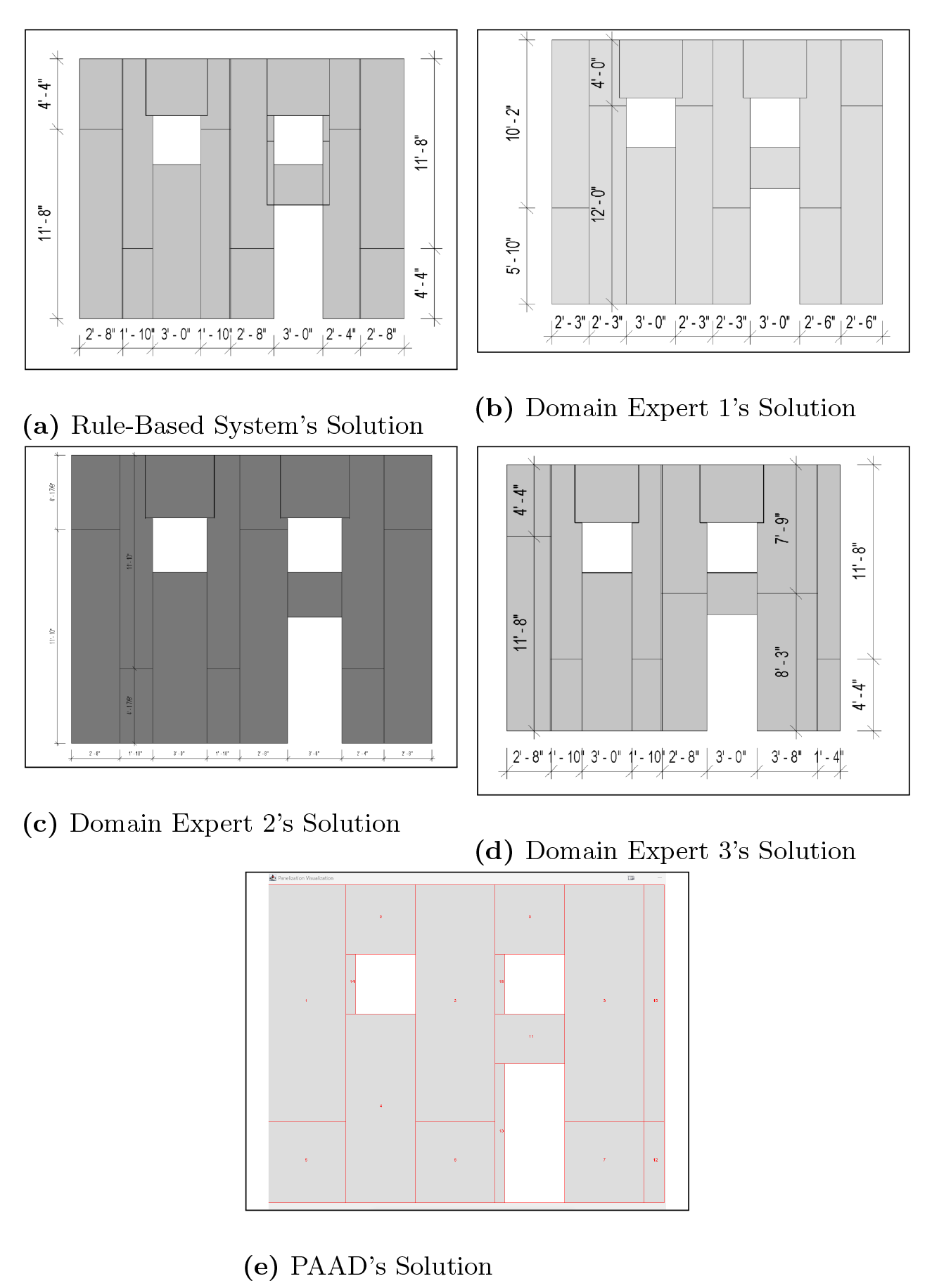

Supplement: S23 Fig — The results of manual scenario 3, where (a) shows the rule-based system’s output, (b)—(d) presents three domain experts’ solutions, and (e) shows PAAD’s solution. (TIF) [file pone.0303646.s023.tif]

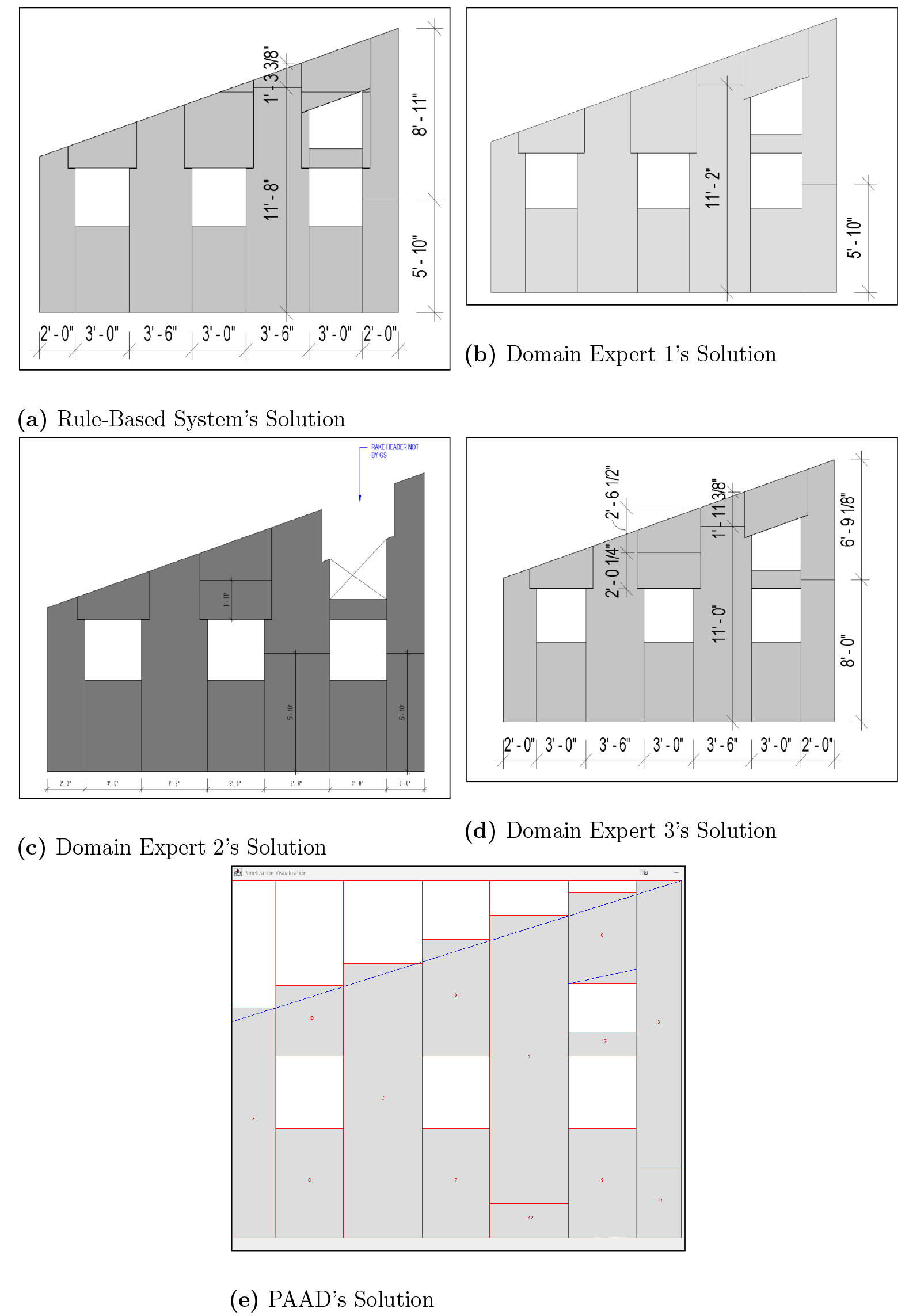

Supplement: S24 Fig — The results of manual scenario 4, where (a) shows the rule-based system’s output, (b)—(d) presents three domain experts’ solutions, and (e) shows PAAD’s solution. (TIF) [file pone.0303646.s024.tif]

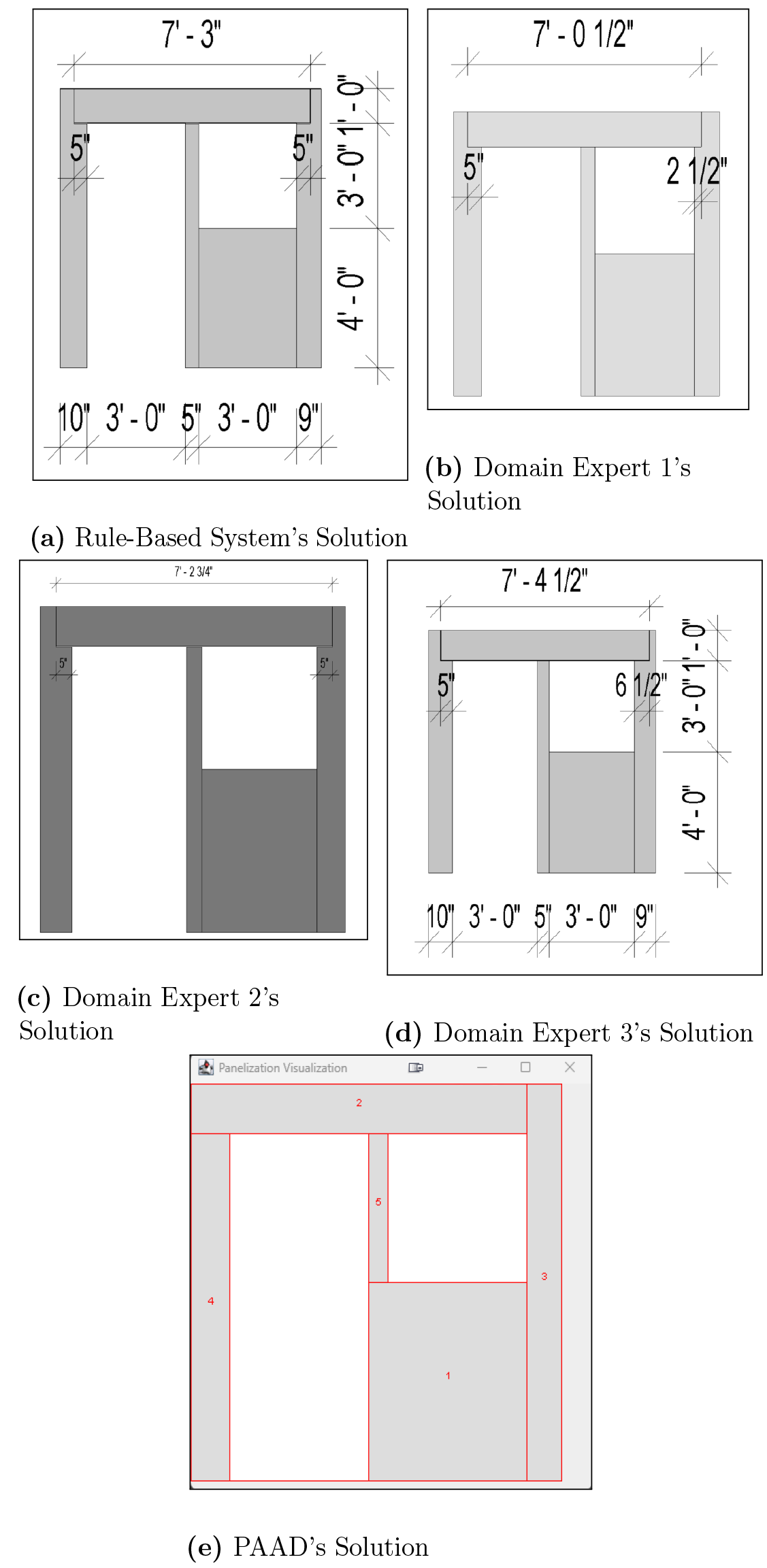

Supplement: S25 Fig — The results of manual scenario 5, where (a) shows the rule-based system’s output, (b)—(d) presents three domain experts’ solutions, and (e) shows PAAD’s solution. (TIF) [file pone.0303646.s025.tif]
