# Supplementary material for: PAAD: Panelization algorithm for architectural designs
Source: PLoS One. 2024 Jun 11;19(6):e0303646. doi: 10.1371/journal.pone.0303646 (PMC11166312; doi:10.1371/journal.pone.0303646)
Supplement: Attachment — Submitted filename: review.docx [file pone.0303646.s026.docx]

The work by Fisher and colleagues presented a novel algorithm called Panelization Algorithm for Architectural Designs (PAAD) to automate the optimal panel configuration. Their approach is built on the 2D bin packing method but engages a genetic evolutionary algorithm. The novelty of this method lies in the capability of adapting to both manufacturing rules (as hard constraints) and structural rules as well as post-processing rules (as flexible constraints) in a flexible way. Comparing against rule-based and domain expert solutions, they showed an overall advantage in PAAD over other methods.

Major issues:

1. Table 1 and 2, the performance of PAAD is compared with rule-based algorithms or domain expert in automated (Table 1) or manual (Table 2) tasks. It is definitely pivotal benchmark to prove the edge of PAAD, however, it might be also interesting to make some visual comparison between the methods in question. I saw such comparisons in Supporting Information, and maybe some can be moved to the main figures to enhance the benchmark.

2. Figures need better captions and description. The figure captions in current manuscript are overly succinct. For example, Fig 4/5, “Scenario 1/2. The results of manual scenario 1/2” but each figure contains five panels (a-e). Detailed description of each subfigure is needed here for the ease of readership.

3. The authors made public the source code at GitHub, which is great. However, I found two issues with the current version. First, the graphical interface has no documentation—only a license can be found at <https://github.com/andrfish/PAAD> at the moment. Second, when I ran the Java program (via the command line java Generator.java), I could only see the wall with random voids above the angled line and stationary openings (please see Review Fig 1-2). However, I could not obtain solutions “superimposed on the architectural drawing” (Line 414). Probably I did not run the program correctly, but this indicates a need for better documentation of the software.

Review Fig 1. The interface output from a run.

Review Fig 2. The interface output from another run.
