# Supplementary material for: PAAD: Panelization algorithm for architectural designs
Source: PLoS One. 2024 Jun 11;19(6):e0303646. doi: 10.1371/journal.pone.0303646 (PMC11166312; doi:10.1371/journal.pone.0303646)
Supplement: Attachment — Submitted filename: PLoS One Rebuttal.docx [file pone.0303646.s027.docx]

Reviewer #1: This research proposes the PAAD algorithm for optimizing panel configurations in architectural designs, addressing inefficiencies in rule-based methods. PAAD utilizes a genetic evolutionary strategy to minimize material waste while complying with manufacturing constraints. Validation against industry implementations and expert solutions demonstrates PAAD's effectiveness and potential to enhance efficiency in the construction industry. The topic of this research is significant.

What are the assumptions for using PAAD algorithm?

Thank you for your positive feedback. We agree that the assumptions concerning our algorithm should be clearly stated, and have now explicitly defined them in the revised manuscript within the methodology section, on page 12/33 in the last paragraph, as follows: *The methodology of PAAD operates under several key assumptions about the problem space: 1) the dimensions of the wall are known, 2) the minimum and maximum sizes of the panels are known, 3) the presence of any angled lines in the wall are known, 4) panels cannot overlap each other, and 5) openings or void spaces must not be covered by panels. We consider these assumptions to not be restrictive for our proposed approach, as they mirror the conditions and practices typically encountered in real-world scenarios.*

Reviewer #2: The work by Fisher and colleagues presented a novel algorithm called Panelization Algorithm for Architectural Designs (PAAD) to automate the optimal panel configuration. Their approach is built on the 2D bin packing method but engages a genetic evolutionary algorithm. The novelty of this method lies in the capability of adapting to both manufacturing rules (as hard constraints) and structural rules as well as post-processing rules (as flexible constraints) in a flexible way. Comparing against rule-based and domain expert solutions, they showed an overall advantage in PAAD over other methods.

Major issues:

1. Table 1 and 2, the performance of PAAD is compared with rule-based algorithms or domain expert in automated (Table 1) or manual (Table 2) tasks. It is definitely pivotal benchmark to prove the edge of PAAD, however, it might be also interesting to make some visual comparison between the methods in question. I saw such comparisons in Supporting Information, and maybe some can be moved to the main figures to enhance the benchmark.

Thank you for the positive feedback. We have incorporated your suggestion to move the “Supporting Information” Figures that were referred to in the “Automated Baseline” to the main manuscript to improve readability.

2. Figures need better captions and description. The figure captions in current manuscript are overly succinct. For example, Fig 4/5, “Scenario 1/2. The results of manual scenario 1/2” but each figure contains five panels (a-e). Detailed description of each subfigure is needed here for the ease of readership.

We appreciate the opportunity to better describe our Figures and improve readability. To address this, nearly all captions have been updated to be more descriptive as well as describe each subfigure where necessary.

3. The authors made public the source code at GitHub, which is great. However, I found two issues with the current version. First, the graphical interface has no documentation—only a license can be found at https://github.com/andrfish/PAAD at the moment. Second, when I ran the Java program (via the command line java Generator.java), I could only see the wall with random voids above the angled line and stationary openings (please see Review Fig 1-2). However, I could not obtain solutions “superimposed on the architectural drawing” (Line 414). Probably I did not run the program correctly, but this indicates a need for better documentation of the software.

Review Fig 1. The interface output from a run.

Review Fig 2. The interface output from another run.

For figures please see the attachment.

To clarify, the original source code available on GitHub included a synthetic data generator and the data definitions for each baseline presented in the manuscript. It did not, however, contain the PAAD code due to agreements with our industry partner, which is why running "Generator.java" only produced a problem space without any solutions. However, following your comment, we discussed the matter with our industry partner, and they have now agreed to allow us to add a JAR file of the PAAD codebase to the repository. We have also enhanced the documentation and added additional code demonstrating how it can be run. We look forward to your verification of our work.

Reviewer #3: The PAAD is very similar to the idea proposed in the following paper, I would suggest explain the difference of your study from the published one.

<https://www.researchgate.net/profile/Hexu-Liu/publication/333907195_Optimizing_multi-wall_panel_configuration_for_panelized_construction_using_BIM/links/5d0bcac5458515c11ceadb66/Optimizing-multi-wall-panel-configuration-for-panelized-construction-using-BIM.pdf>

Thank you for providing this study. We have reviewed it and believe that our comments towards the end of the "Related Works" section emphasize the unique aspects of PAAD, notably its basis in genetic evolution rather than being merely a mathematical model. Nonetheless, we have cited the study at the beginning of the "Related Works" section. Furthermore, an explicit statement has been added at the end of this section to outline the novelties of our proposed work (1) generative design techniques for panelization, 2) the integration of manufacturing constraints within the design framework, 3) improved explainability via simple problem definitions and a preliminary interface, and 4) the ability to add post-processing rules that reflect revisions typically made by domain experts) as follows: *This represents a distinction over traditional mathematical models and BIM-generative design frameworks, as PAAD offers greater flexibility in generating solutions while still adhering to the complexities of the problem space without needing explicit optimization definitions*. Additionally, we have incorporated visuals of the different panel types considered by PAAD in the "Constraints" section to highlight another distinction, now referred to towards the end of the first paragraph there.

There are 4 algorithms included in the manuscript. And also, one Github link is provided. However, hard to connect the 4 algorithms with the Github Repo. There is no README file in the Github. Please make your data and scripts easy to follow.

We appreciate the opportunity to clarify the contents of our GitHub repository. Initially, it was solely a data repository, containing scripts for synthetic data generation and the definitions used in our baseline analyses. The source code for PAAD was not included due to an agreement with our industry partner. However, following your comments and subsequent discussions with our partner, we have now received permission to add a compiled JAR file of the PAAD algorithm to the repository. Additionally, we have enhanced the documentation and included extra code to demonstrate how it can be run. We look forward to having you verify our work.

Please add more details of explaining each figure. I only see the figure title.

We appreciate the opportunity to better describe our Figures and improve readability. To address this, nearly all captions have been updated to be more descriptive as well as describe each subfigure where necessary. As per PLoS One submission guidelines, the main manuscript’s images are submitted as separate files during the review process rather than included in the PDF.

Reviewer #4: Please see my comments/questions below:

Figures are not well labeled. When I read this manuscript, I had a hard time to figure out which figures are which.

We appreciate the opportunity to better describe our Figures and improve readability. To address this, nearly all captions have been updated to be more descriptive as well as describe each subfigure where necessary.

The abbreviation 2DBP is introduced in line 36, so you can directly use it in line 154.

For equation (1) and (2), I would suggest to use left alignment.

Thank you for these comments on abbreviating and formatting, we have incorporated the suggested changes.

I don’t understand equation (3), which does't look like a traditional math equation. Could you please explain it? For example, it seems that “fitness(P,W,V,O)” is a superscript of “P,W,O,V,M,C”, what does it mean? What are the subject of “minimize” and “subject to” in this equation?

To clarify, this equation defines an optimization function to summarize the objective that PAAD aims to achieve, using traditional optimization formulae. The variables are described right after the equation, and we have reiterated the algorithmic definitions in the revised manuscript to improve readability as follows: *where P is a set of population objects p that were each initialized using Algorithm 1, W is the wall definition (i.e., height and width), O is the openings definition (i.e., where each opening has a height, width, starting x-coordinate, and starting y-coordinate), V is the void area definitions (i.e., for space above angled lines), M is a mutation probability, C is a crossover probability, fitness represents a function that evaluates Eq 2 for each p in P to determine the minimum result, and evolve_panels represents Algorithm 2. To summarize their definitions from before, Algorithm 1 initializes populations by generating potential panelization solutions within manufacturing constraints, Eq 2 assesses each population's fitness with the aim of covering a wall using the fewest panels, and Algorithm 2 evolves these populations through mutations that alter panel dimensions and crossovers that merge potential solutions.*
